# Deletion of the Histone Deacetylase HdaA in Endophytic Fungus *Penicillium chrysogenum* Fes1701 Induces the Complex Response of Multiple Bioactive Secondary Metabolite Production and Relevant Gene Cluster Expression

**DOI:** 10.3390/molecules25163657

**Published:** 2020-08-11

**Authors:** Zhuang Ding, Haibo Zhou, Xiao Wang, Huiming Huang, Haotian Wang, Ruiyan Zhang, Zhengping Wang, Jun Han

**Affiliations:** 1 Institute of BioPharmaceutical Research, Liaocheng University, Liaocheng 252059, China; wangxiao1mail@163.com (X.W.); zry147896@163.com (R.Z.); wangzhengping@lcu.edu.cn (Z.W.); hanjun@lcu.edu.cn (J.H.); 2 Shandong University-Helmholtz Institute of Biotechnology, State Key Laboratory of Microbial Technology, Shandong University, Qingdao 266237, China; haibozhou@sdu.edu.cn; 3 School of Life Science, Liaocheng University, Liaocheng 252059, China; huanghuiming@lcu.edu.cn; 4 Faculty of Pharmacy, Bengbu Medical College, Bengbu 233000, China; haotian@bbmc.edu.cn

**Keywords:** metabolic regulation, roquefortine, meleagrin, endophytic fungi, *Penicillium chrysogenum*

## Abstract

Epigenetic regulation plays a critical role in controlling fungal secondary metabolism. Here, we report the pleiotropic effects of the epigenetic regulator HdaA (histone deacetylase) on secondary metabolite production and the associated biosynthetic gene clusters (BGCs) expression in the plant endophytic fungus *Penicillium chrysogenum* Fes1701. Deletion of the *hdaA* gene in strain Fes1701 induced a significant change of the secondary metabolite profile with the emergence of the bioactive indole alkaloid meleagrin. Simultaneously, more meleagrin/roquefortine-related compounds and less chrysogine were synthesized in the *ΔhdaA* strain. Transcriptional analysis of relevant gene clusters in *ΔhdaA* and wild strains indicated that disruption of *hdaA* had different effects on the expression levels of two BGCs: the meleagrin/roquefortine BGC was upregulated, while the chrysogine BGC was downregulated. Interestingly, transcriptional analysis demonstrated that different functional genes in the same BGC had different responses to the disruption of *hdaA*. Thereinto, the *roqO* gene, which encodes a key catalyzing enzyme in meleagrin biosynthesis, showed the highest upregulation in the *ΔhdaA* strain (84.8-fold). To our knowledge, this is the first report of the upregulation of HdaA inactivation on meleagrin/roquefortine alkaloid production in the endophytic fungus *P. chrysogenum*. Our results suggest that genetic manipulation based on the epigenetic regulator HdaA is an important strategy for regulating the productions of secondary metabolites and expanding bioactive natural product resources in endophytic fungi.

## 1. Introduction

Filamentous fungi are well-known producers of diverse secondary metabolites (SMs), which have a wide range of biological activities and can be beneficial or harmful to human beings [1,2]. On the one hand, many beneficial fungi-derived SMs have long been clinically utilized as antibacterials (penicillin and cephalosporin), antifungals (anidulafungin and caspofungin), immunosuppressants (cyclosporin), and antihypercholesterolemic drugs (lovastatin) [3]. On the other hand, some mycotoxins can severely endanger public health, such as aflatoxins, fumonisins, fusarins, and gliotoxin [4]. As an industrial microbial strain used for producing the β-lactam antibiotic penicillin, *Penicillium chrysogenum* has the capacity to synthesize many SMs with diverse chemical structures and significant bioactivities, such as alkaloids, polyketides, and terpenoids [5,6,7]. In recent decades, genome sequencing and genetic studies of *P. chrysogenum* have led to the elucidation of many secondary metabolic pathways for SM production [8,9]. Subsequently, the functions of some transcription regulators associated with these biosynthetic pathways have been well studied. For instance, the regulatory factors PcRFX1 and PcFKH1 have been characterized to positively control penicillin biosynthesis in *Penicillium chrysogenum* [10,11]. Similarly, the deletion of the transcription factor gene *laeA* resulted in a drastic decrease of penicillin gene expression [12]. In contrast, another transcription factor CreA was reported to negatively regulate penicillin production [13].

Recent studies have shown that the secondary metabolism of filamentous fungi is controlled by a complicated and elaborate regulatory network, which is influenced by not only various transcription factors but also epigenetic regulators [14]. Among various types of epigenetic regulators in eukaryotic cells, histone deacetylases (HDACs) play an important role and profoundly influence DNA replication, transcription, and repair processes [15]. Furthermore, several reports have determined that histone deacetylation induced by HDACs tends to be associated with heterochromatin and gene silencing [16]. Thus, the inactivation of HDACs has been widely considered as an available strategy for the activation of silent SM biosynthetic pathways and increasing the production of bioactive natural products in filamentous fungi. Shwab et al. found that the deletion of the HDAC-encoding gene *hdaA* resulted in the transcriptional activation of penicillin and sterigmatocystin biosynthetic gene cluster (BGC) in *Aspergillus nidulans* strain A89 [17]. Deletion of the homologue of *hdaA* in *Aspergillus fumigatus* strain AF293 increased the transcription of several NRPS (nonribosomal peptide synthetase) gene clusters [18]. By disrupting *hdaA* in *Calcarisporium arbuscular* NRRL3705, Mao et al. activated 75% of the SM biosynthetic genes and found four new compounds [19]. Moreover, the deletion of *hdaA* in *Pestalotiopsis fici* CGMCC3.15140 activated the production of a series of macrodiolides [20]. In addition, it was found that HDACs could also regulate the transcription of the genes that were associated with conidiation, sexual reproduction, growth, stress response, and pathogenicity [21].

As a characteristic bioresource, endophytic fungi provide a broad variety of important SMs with diverse bioactivities [22,23]. These SMs are not only relevant to the physiological and ecological peculiarity of fungi, but are also interrelated with human life and health. Recent investigations on endophyte genomes revealed diverse secondary metabolic BGCs [19,24]. However, the considerable biosynthetic potential of endophytic fungi is a reflection of the complex ecological environment, which is difficult to simulate under laboratory conditions, resulting in most BGCs being silent [19]. According to the aforementioned reports, genetic manipulation based on HDAC genes represents a feasible strategy for regulating and activating secondary metabolic BGCs in eukaryotes. However, the application of this regulation technique is limited to endophytic fungi. In this study, we constructed an *hdaA* homologue deletion mutant of *P. chrysogenum* Fes1701, an endophytic fungus isolated from rubber tree (*Ficus elastica*) leaves [25], and investigated the effects of HdaA inactivation on secondary metabolism. Our results indicate that the *ΔhdaA* strain showed a significant change of secondary metabolic profile with the emergence of some SMs. In addition, we describe the effects of HdaA inactivation on the expression of two SM-associated BGCs of *P. chrysogenum* Fes1701.

## 2. Results and Discussion

### 2.1. Identification and Deletion of the hdaA Gene in P. chrysogenum Fes1701

**Figure 1:**
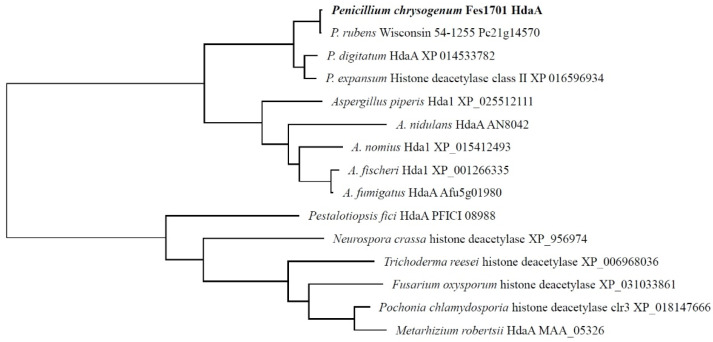
Phylogenetic tree analyses of HdaA in the strain Fes170 and its homologs from different species. Branch lengths are in proportion to distance.

In the whole genomic sequence of *P. chrysogenum* Wis54-1255, the *hdaA* gene is located in chromosome 2 and is designated as Pc21g14570 [8]. The *hdaA* gene of the strain Fes1701 was located and identified in the genomic sequence via Local-BLAST. Subsequently, we designed special primers to clone the *hdaA* ORF from the genomic DNA of the strain Fes1701. The obtained PCR fragment was 3039 bp in size, and the predicted coding sequence was 2304 bp, which encoded a 767 amino acid polypeptide. To confirm its identity, bioinformatics analysis of HdaA was performed using an NCBI BLAST search. BLAST analysis indicated that this protein showed 66.8% sequence identity to HdaA (AN8042) of *Aspergillus nidulans* [26]. The taxonomic relatedness of HdaA and other known homologous protein sequences from other species is shown by a phylogenetic tree in Figure 1. In addition, reverse transcription PCR was performed to determine the transcription level of *hdaA*. Results indicated that the *hdaA* transcript was normally expressed when the wild type (WT) of the Fes1701 strain was grown at 25 °C on PDA (potato dextrose agar) medium (Figure S1).

**Figure 2:**
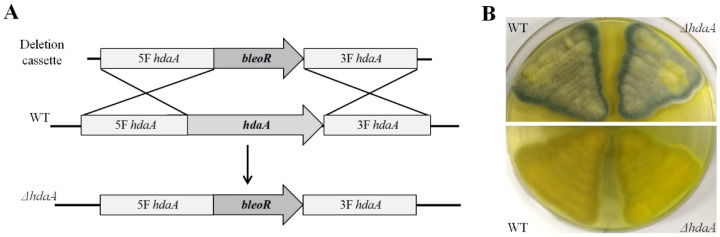
Generation and phenotype of the Δ*hdaA* strain. (**A**) Schematic illustration for *hdaA* disruption. The *bleoR* gene is amplified from the plasmid pZeo, and the bleomycin is used for the selection of transformants bearing the *bleoR* gene. Transformation was performed by homologous recombination using the protoplast transformation method. (**B**) The phenotype of the *ΔhdaA* and WT strains grown on PDA plates (25 °C for 5 days).

Upstream and downstream flanking fragments of the *hdaA* ORF were PCR-amplified for the construction of a gene disruption cassette (Table S1). An *hdaA* deletion mutant (*ΔhdaA*) was constructed by replacing this gene with a bleomycin resistance gene (*bleoR*) cassette (Figure 2A). A 0.1% bleomycin-supplemented medium was used for the positive selection of the transformant. The genomic DNA of the selected transformant was extracted and further verified using diagnostic PCR (Figure S2). The 1.9-kb and 2.1-kb fragments could be amplified from the correct *ΔhdaA* strain using the primers VP1–VP5 and VP2–VP6, respectively, but were absent in the WT. No PCR product was amplified from the *ΔhdaA* strain using primers VP3–VP4, while a 1.7-kb product could appear using the genomic DNA of the WT (Figure S2). The WT and *ΔhdaA* strains were cultured on PDA medium at 25 °C for phenotype observation. Comparative results indicate that there was no significant difference in phenotype between the WT and *ΔhdaA* strains (Figure 2B).

### 2.2. Effects of hdaA Disruption on SMs Production

**Figure 3:**
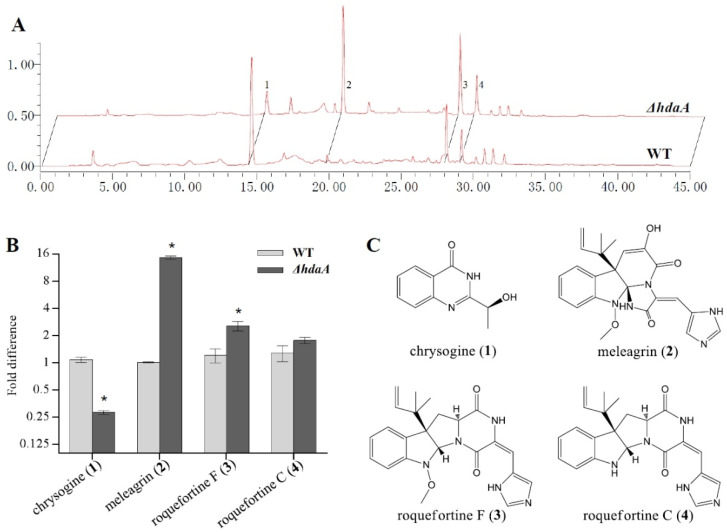
Secondary metabolite profiles of *ΔhdaA* and WT strains. (**A**) HPLC analysis of secondary metabolite profiles. (**B**) Relative amounts of four main products 1–4 in Δ*hdaA* compared with the WT. Mean values with asterisks are significant. (**C**) The chemical structure of the four main products detected in this study: 1, chrysogine; 2, meleagrin; 3, roquefortine C; 4, roquefortine F. The analysis for each strain was performed in triplicate. Mean values with asterisk are significant at *p* < 0.01.

To examine the effect of *hdaA* deletion on secondary metabolism in the strain Fes1701, the SMs extract of the *ΔhdaA* strain was analyzed using HPLC and compared with that of the WT. After cultivating in PDB (potato dextrose broth) medium for 5 days, the metabolite fingerprints showed obvious differences between the *ΔhdaA* and WT strains (Figure 3A,B). Four main SMs (1−4) in the HPLC fingerprint of the *ΔhdaA* strain were further purified for the identification of their chemical structures (Figure 3C). By comparison with the published MS and NMR data in the literature [27,28,29,30], these compounds were identified as chrysogine (1), meleagrin (2), roquefortine F (3), and roquefortine C (4) (Figures S3–S10). The productions of four compounds in the *ΔhdaA* and WT strains were comparatively analyzed by calculating the HPLC peak area. Results showed that chrysogine (1) in the *ΔhdaA* strain had a 3.8-fold decrease as compared with the WT. Simultaneously, the production of compounds 2−4 showed 14.4-, 2.1-, and 1.4-fold increases in the *ΔhdaA* strain (Figure 3B). Similar multiple effects of HDAC on the production of SMs have been found in other fungal species [18,31]. For instance, Lee et al. reported that deletion of the *hdaA* gene in *A. fumigatus* increased the production of several SMs but decreased the production of gliotoxin [18].

### 2.3. Effects of hdaA Disruption on the Transcription of SM Biosynthetic Gene Clusters

**Figure 4:**
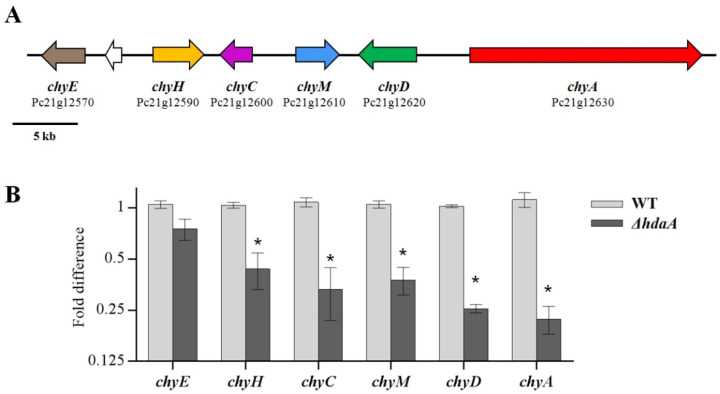
Transcriptional analysis of the chrysogine biosynthetic gene cluster. (**A**) Organization of the chrysogine biosynthetic gene cluster (BGC). (**B**) Quantitative RT-PCR analysis of the chrysogine BGC. The analysis for each strain was performed in triplicate. Data are shown as fold change relative to the first trial of the WT. Mean values with asterisk are significant at *p* < 0.01.

Because the deletion of *ΔhdaA* greatly influenced the production of chrysogine (1) and meleagrin (2), we further investigated the differences in the transcriptional levels of genes from two BGCs encoding the enzymes responsible for the production of these two compounds between the *ΔhdaA* and WT strains. Chrysogine (1) is a yellow alkaloid produced by several filamentous fungi, such as the genera *Penicillium, Aspergillus*, and *Fusarium* [32]. Although this compound was first isolated in 1973, its BGC was not clarified until 2017 [32,33]. Viggiano et al. [33] elucidated the chrysogine biosynthetic pathway in *P. chrysogenum* and identified the function of each enzyme involved in this pathway. The *P. chrysogenum* chrysogine biosynthetic pathway contains a NRPS (Pc21g12630) flanked by five associated genes (Pc21g12570, Pc21g12590, Pc21g12600, Pc21g12610, and Pc21g12620), designated as *chyA* to *chyH* (Figure 4A). In our study, the transcriptional levels of these genes contained in the chrysogine BGC in *ΔhdaA* were significantly downregulated as compared with the WT (Figure 4B). Thereinto, the transcriptional levels of Pc21g12630 (*chyA*), which encodes an NRPS, and Pc21g12620 (*chyD*), which encodes an amidase, showed a 4.0-fold and 5.0-fold decrease in *ΔhdaA*, respectively. A similar phenomenon was observed by Guzman-Chavez et al. [34], who also reported that the inactivation of HdaA caused the downregulation of chrysogine BCG expression in the industrial strain *P. chrysogenum* DS68530.

**Figure 5:**
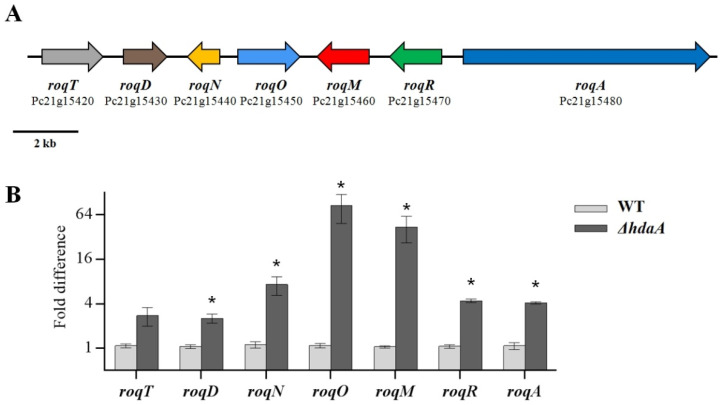
Transcriptional analysis of the meleagrin/roquefortine biosynthetic gene cluster. (**A**) Organization of the meleagrin/roquefortine BGC. (**B**) Quantitative RT-PCR analysis of the meleagrin/roquefortine BGC. The analysis for each strain was performed in triplicate. Data are shown as fold change relative to the first trial of the WT. Mean values with asterisk are significant at *p* < 0.01.

In *P. chrysogenum*, the meleagrin/roquefortine BGC is a seven-gene cluster extending from Pc21g15420 to Pc21g15480 (Figure 5A). It encodes proteins from the biosynthetic pathway where, as was confirmed, roquefortine C is a biosynthetic precursor of meleagrin [35]. The biosynthesis of roquefortine depends on the involvement of the upstream genes, including the Pc21g15420 (*roqT*), Pc21g15430 (*roqD*), Pc21g15440 (*roqN*), and Pc21g15480 (*roqA*) genes. Subsequently, the Pc21g15450 (*roqO*) and Pc21g15460 (*roqM*) genes, which respectively encode a P450 scaffold reorganizing oxygenase and a MAK1 monooxygenase, are responsible for the biotransformation from roquefortine to meleagrin [35]. In our study, a comparative analysis of the transcriptional levels showed that these upstream genes responsible for roquefortine were 2.5- to 7.5-fold upregulated in *ΔhdaA* (Figure 5B). Furthermore, most significant enhancements appeared downstream of this pathway, which were essential for the biosynthesis of meleagrin. In this stage, Pc21g15450 (*roqO*) and Pc21g15460 (*roqM*) showed an 84.8- and 43.7-fold increase in *ΔhdaA* (Figure 5B). This phenomenon was relevant to the production increase of the meleagrin/roquefortine BGC in the Δ*hdaA* strain. The productions of roquefortine and related compounds exhibited a significant increase, while meleagrin appeared in the metabolite profiling of the Δ*hdaA* strain as a newly generated compound (Figure 3A). In the previous report, the production of meleagrin/roquefortine-related compounds in *P. chrysogenum* were unaffected by the inactivation of HdaA [34]. However, the production of these compounds in the *ΔhdaA* strain were significantly increased in this study. This phenomenon could indicate that the secondary metabolic pathway in the same fungal species derived from different habitats may be affected synergistically by other additional regulatory mechanisms. For example, a heterotrimeric Ga protein Pga1 has been reported to upregulate the biosynthesis of roquefortine in *P. chrysogenum* Wis54-1255 [36]. Our results demonstrate that it is important to reveal the different effects of epigenetic regulators in the same organism on secondary metabolism.

### 2.4. Bioactivities of the Metabolites Isolated from the ΔhdaA Strain

The meleagrin and roquefortine groups have been reported to possess various biological properties, such as antibacterial, neurotoxic, cytochrome P450 inhibitory, and tubulin polymerization inhibitory activities [37]. Because of the increasing production of meleagrin and roquefortine compounds (2–4) in the *ΔhdaA* strain, their antimicrobial and cytotoxic activities were further evaluated in this study (Table 1). Meleagrin (2) exhibited the best growth-inhibitory activity against two different leukemia cells (K562 and HL-60) with IC_50_ values of 8.9 and 12.7 μM. A similar level of antineoplastic activity was reported by Mady et al. [38], in which meleagrin (2) showed significant inhibitory activities against the proliferation and migration of c-Met-dependent breast malignancy. Roquefortine alkaloids (3,4) showed a medium antibacterial activity against Gram-negative bacterium *Escherichia coli* and Gram-positive bacterium *Staphylococcus aureus*. The inhibitory activity of roquefortine alkaloids against Gram-positive bacteria have been reported [39], especially the antibacterial activity of roquefortine C (4) against *Bacillus subtilis* with an MIC of 6.3 µg/mL [40]. However, all tested compounds did not show obvious antifungal activity in two pathogenic yeast fungi (MIC > 128 μg/mL).

## 3. Materials and Methods

### 3.1. Strains and Media

The *Penicillium chrysogenum* Fes1701 wild type (WT) was originally isolated from fresh leaves of *Ficus elastica* collected in Liaocheng University Arboretum, Liaocheng, China [24]. The WT and its transformants were grown on PDA medium at 25 °C, 5 days for sporulation, and the fresh spores were harvested with 0.01% Tween 80. The *Escherichia coli* strain DH5α was cultured in Luria–Bertani liquid medium at 37 °C with 100 µg/mL ampicillin for plasmid propagation.

### 3.2. Cloning and Identification of the hdaA Gene in Strain Fes1701

Fungal genomic DNA was isolated using the CTAB method [25]. The oligonucleotide sequences of PCR primers are listed in Table S1. The HdaA protein sequence of *P. chrysogenum* Wisconsin 54-1255 (Pc21g14570) was used as query to perform BLAST searches to find its ortholog in the strain Fes1701. The genome sequence of *P. chrysogenum* Wisconsin 54-1255 was obtained from the European Molecular Biology Laboratory (EMBL) (Heidelberg, Germany) under accession numbers AM920416–AM920464. The designed PCR primers PchdaA-F and PchdaA-R were used to amplify the *hdaA* ORF from the genomic DNA of strain Fes1701 using the TransStart^®^ FastPfu DNA Polymerase (TransGen Biotech, Beijing, China). A 3.0-kb fragment was obtained and subcloned using a pEasy^®^ Blunt Cloning Kit (TransGen Biotech, Beijing, China) to yield pEasy-PcHdaA and sequenced by Sangon Biotech (Shanghai, China). The sequence data of the *hdaA* gene in strain Fes1701 were deposited in the GenBank Database under accession number MT313928. The coding sequence for amino acid polypeptide was predicted by the FGenesh software (Softberry Inc., NY, USA) [41]. Phylogenetic analysis of HdaA orthologs from strain Fes1701 and other fungi was performed using the MEGA 7.0 software [42].

### 3.3. Creation of the ΔhdaA Strain

Deletion of the *hdaA* gene in the WT was performed through homologous recombination. A gene disruption cassette was assembled using the fusion PCR method as described previously [43] and inserted into pEasy-Blunt Cloning Vector (TransGen Biotech) to gain the plasmid pEB-HDD. The gene disruption cassette was comprised of an upstream flanking region (2.2 kb, amplified by the primer ΔHdaA-P1 and P2), bleomycin resistance gene (*bleoR*, 1.5 kb, amplified from the plasmid pZeo [43] by the primer ΔHdaA-P3 and P4), and downstream flanking region (1.9 kb, amplified by the primer ΔHdaA-P5 and P6), consecutively. The disruption cassette was excised from pEB-HDD with the restriction enzymes KpnI and PacI and then transferred into the WT, yielding the *ΔhdaA* strain by PEG-mediated protoplast transformation. Transformants were selected and single-spore purified in the presence of bleomycin. The genotype of the deletion mutant was confirmed by diagnostic PCR.

### 3.4. Fermentation Conditions and SM Extraction

For SMs production, the *ΔhdaA* and WT strains were cultured in PDB medium. The cultures were inoculated with 1 × 10^5^ fresh spores in 250 mL flasks containing 100 mL of PDB medium and grown under shaking condition at 180 rpm, 25 °C for 5 days. Each test contained three replicates for each strain.

The extraction of fermentation products was performed following the protocols established by Ding et al. [25]. After cultivation, the mycelia in the fermentation mixture were broken using a disperser (T18, IKA, Germany), then the fermentation mixture was extracted with an equal volume of ethyl acetate. The extract was evaporated under reduced pressure and redissolved in 2 mL MeOH. Sterile PDB medium as the control was extracted using the same procedure.

### 3.5. Metabolite Fingerprint Analysis

A 10 µL extract was injected into the HPLC system (Waters Inc., Milford, MA, USA), which contained a model 1525 pump, an ODS column (Pack ODS-A, 250 × 4.6 mm, 5 µm, YMC Co., Ltd., Japan), and a model 2489 UV detector. The gradient increased from 20% to 100% MeOH over 30 min and then was retained at 100% MeOH for 10 min. The fold differences of these compounds between the mutant and WT were calculated by HPLC peak area according to the following formula: [Area (Sample)—Area (Control)]/[Area (WT)–Area (Control)].

### 3.6. Purification and Identification of Natural Products

A two-liter-scale culture and extract preparation of the *ΔhdaA* strain were performed using the method mentioned above. The obtained extract (1.3 g) was gained and separated by silica gel vacuum liquid chromatography using MeOH-H_2_O to give four fractions (Fractions 1–4). Fraction 1 was further separated by Sephadex LH-20 eluted with MeOH and then on a semipreparative HPLC column (Pack ODS-A, 250 × 10 mm, 5 µm, YMC Co., Ltd., Japan) eluted with MeOH-H_2_O (50:50, 3 mL/min) to provide compound **1** (2.6 mg, *t*_R_ 8.5 min). Fraction 2 was separated by semipreparative HPLC eluted with MeOH-H_2_O (60:40, 3 mL/min) to obtain compound **2** (5.7 mg, *t*_R_ 10.5 min). Fraction 4 was separated on a Sephadex LH-20 column with CH_2_Cl_2_-MeOH (1:1) and then on a semipreparative HPLC eluted with MeOH-H_2_O (75:25, 3 mL/min) to obtain compound **3** (3.5 mg, *t*_R_ 11.5 min) and compound **4** (3.6 mg, *t*_R_ 12.5 min). The structures of the compounds were identified using MS and NMR data. MS spectra were recorded on a Q-TOF Ultima Global GAA076 LC mass spectrometer (Wasters Inc., Milford, MA, USA). NMR spectra were collected on a Varian 500 spectrometer (Varian Medical Systems Inc., Palo Alto, CA, USA).

### 3.7. RNA Extraction and Real-Time PCR Analysis

The relative expression levels of the chrysogine and roquefortine/meleagrin biosynthetic gene clusters in the *ΔhdaA* and WT strains were analyzed by real-time PCR. The mutant and WT were cultivated in PDB medium as the abovementioned fermentation condition. The mycelia of each strain were collected on the 5th day, and RNA was extracted from the mycelia using the EasyPure^®^ RNA kit for qRT-PCR (ER101, TransGen Biotech, China) following the manufacturer’s protocol. Then, the quality of the RNA was checked by a NanoDrop 2000 nucleotide analyser (Thermo Scientific, Waltham, MA, USA). cDNA was synthesized using the TransScript^®^ kit (AH341, TransGen Biotech, China). Real-time PCR was performed using a CFX96 Real-Time System (Bio-Rad, Hercules, CA, USA) using the TransStart^®^ kit (AQ132, TransGen Biotech, China). The PCR reaction system were 10 μL 2 × qPCR mix, 0.5 μL forward/reverse primer (10 μM), 1 μL template cDNA, and water to 20 μL. The PCR reaction conditions were 95 °C for 2 min, 45 cycles of 95 °C for 5 s, 60 °C for 15 s, 72 °C for 25 s, and 95 °C for 10 s. Three replicates of each cDNA sample were carried out, and the average threshold cycle was calculated. Relative expression levels were calculated using the 2^−ΔΔCt^ method with the expression level of the actin gene as a reference. The primers used for real-time PCR are listed in Table S1.

### 3.8. Bioactivity Assay

Cytotoxicity against two human tumor cell lines, K562 (human chronic myeloblastic leukemia cells) and HL-60 (human promyelocytic leukemia cells), was evaluated using the methyl-thiazolyl-tetrazolium (MTT) method with Adriamycin as positive control [44].

Antimicrobial activities against four microorganisms, including the Gram-negative bacterium *Escherichia coli* CMCC 44102, the Gram-positive bacterium *Staphylococcus aureus* CMCC 26003, the fungus *Candida albicans* ATCC 10231, and *C. glabrata* ATCC 15126, were performed using the sequential 2-fold dilutions method as previously reported [45]. Chloramphenicol and fluconazole were used as the positive controls for antibacterial and antifungal assays, respectively.

### 3.9. Statistical Analysis

Comparisons of results were analyzed using the GraphPad software (version 7.01) (GraphPad software, San Diego, CA, USA) followed by the Tukey–Kramer test at *p* < 0.01. Values with asterisks are statistically significant.

## 4. Conclusions

As a characteristic bioresource, endophytic microorganisms have been increasingly recognized as a significant reservoir for exploiting bioactive SMs [46]. With the availability of a huge number of fungal genome sequences, SM BGCs and various metabolic regulators associated with these BGCs have become one of the research hot spots in fungal chemical biology. However, the identification of epigenetic regulators and their effects on secondary metabolism is still limited, especially in endophytic fungi. In the present study, we constructed an *hdaA* deletion mutant of the endophytic fungus *P. chrysogenum* Fes1701 and verified its effects on secondary metabolism. SMs profile analysis showed that the *ΔhdaA* strain produced less chrysogine- and more roquefortine-related compounds. Transcriptional analysis demonstrated that the deletion of *hdaA* activated the expression of the meleagrin/roquefortine BGC but inhibited the expression of the chrysogine BGC. Results from the current study suggest that the genetic manipulation of the epigenetic regulator HdaA represents a promising approach for activating and promoting yields of bioactive compounds in endophytic fungi.

## Figures and Tables

**Table 1:** Bioactivities of the meleagrin and roquefortine compounds from the *ΔhdaA* strain.

Compound	Antimicrobial Activity (MIC, μg/mL)				Cytotoxicity (IC_50_, μM)	
	*E. coli*	*S. aureus*	*C. albicans*	*C. glabrata*	K562	HL-60
Meleagrin	128	32	>128	>128	8.9	12.7
Roquefortine C	64	16	>128	>128	27.4	28.1
Roquefortine F	64	16	>128	>128	22.7	25.1
Chloramphenicol	2	1	n.t.	n.t.	n.t.	n.t.
Fluconazole	n.t.	n.t.	1	1	n.t.	n.t.
Adriamycin	n.t.	n.t.	n.t.	n.t.	0.3	0.1

Antibacterial chloramphenicol, antifungal fluconazole, and anticancer Adriamycin were used as positive control. n.t.–not tested.

## Supplementary Materials

Table S1: Primers used in this study; Figure S1: Transcriptional analysis of a potential *hdaA* regulator gene by reverse transcription PCR; Figure S2: PCR verification of the *ΔhdaA* strain; Figures S3–S6: 1H-NMR spectrum of compounds 1–4 (*CH_3_Cl-d_3_*); Figures S7–S10: MS spectrum of compounds 1–4.
