# Supplementary material for: Deletion of the Histone Deacetylase HdaA in Endophytic Fungus Penicillium chrysogenum Fes1701 Induces the Complex Response of Multiple Bioactive Secondary Metabolite Production and Relevant Gene Cluster Expression
Source: Molecules. 2020 Aug 11;25(16):3657. doi: 10.3390/molecules25163657 (PMC7464707; doi:10.3390/molecules25163657)
Supplement: Supplementary file 1 [file molecules-25-03657-s001.pdf]

Supplementary Material

Deletion of the Histone Deacetylase HdaA in Endophytic Fungus *Penicillium chrysogenum* Fes1701 Induces the Complex Response of Multiple Bioactive Secondary Metabolite Production and Relevant Gene Cluster Expression

Zhuang Ding [1,*], Haibo Zhou [2], Xiao Wang [1], Huiming Huang [3], Haotian Wang [4], Ruiyan Zhang [1], Zhengping Wang [1] and Jun Han [1]

[1] Institute of BioPharmaceutical Research, Liaocheng University, Liaocheng, Shandong 252059, China; wangxiao1mail@163.com (X.W.); zry147896@163.com (R.Z.); wangzhengping@lcu.edu.cn (Z.W.); hanjun@lcu.edu.cn (J.H.)
[2] Shandong University-Helmholtz Institute of Biotechnology, State Key Laboratory of Microbial Technology, Shandong University, Qingdao, Shandong 266237, China; haibozhou@sdu.edu.cn
[3] School of Life Science, Liaocheng University, Liaocheng, Shandong 252059, China; huanghuiming@lcu.edu.cn
[4] Faculty of Pharmacy, Bengbu Medical College, Bengbu, Anhui 233000, China; haotian@bbmc.edu.cn
* Correspondence: dingzhuang@lcu.edu.cn; Tel./Fax: +86-635-8239136

Table S1. Primers used in this study

Figure S1. Transcription analysis of potential *hdaA* regulator gene (Pc21g14570) by reverse-transcription PCR.

Figure S2. PCR verification of *ΔhdaA* strain.

Figure S3. ^{1}H-NMR spectrum of compound **1** (CH_3Cl-d_3).

Figure S4. ^{1}H-NMR spectrum of compound **2** (CH_3Cl-d_3).

Figure S5. ^{1}H-NMR spectrum of compound **3** (CH_3Cl-d_3).

Figure S6. ^{1}H-NMR spectrum of compound **4** (CH_3Cl-d_3).

Figure S7. MS spectrum of compound **1**.

Figure S8. MS spectrum of compound **2**.

Figure S9. MS spectrum of compound **3**.

Figure S10. MS spectrum of compound **4**.

Table S1. Primers used in this study.

Primer Name	Template Gene [a]	Sequence 5'-3'	Function
PchdaA-F	Pc21g14570		*hdaA* cloning
PchdaA-R	Pc21g14570		
PchdaA-TF	Pc21g14570	TGACTGGTCTGACGAGGAGG	*hdaA* transcription analysis
PchdaA-TR	Pc21g14570	GCGACTGCAGAGTCAGTCGG	
Actin F	Actin	CTCTGCCCCACGCTATCTCG	
Actin R	Actin	ACGATGGAAGGACCGCTCTC	
ΔHdaA-P1	Pc21g14570 (upstream)	CCTTCTTCGCTGAGCGTGACCCCGCTGCCATCCCCTGGGG	*hdaA* deletion
ΔHdaA-P2	Pc21g14570 (upstream)	TCAATATCATCTTCTGTCGACATGCAAGGGAAAGCCACGG	
ΔHdaA-P3	BleoR	CCGTGGCTTTCCCTTGCATGTCGACAGAAGATGATATTGA	
ΔHdaA-P4	BleoR	GGAATCAAGAAATCAAGGAAAAGAAGGATTACCTCTAAAC	
ΔHdaA-P5	Pc21g14570 (downstream)	GTTTAGAGGTAATCCTTCTTTTCCTTGATTTCTTGATTCC	
ΔHdaA-P6	Pc21g14570 (downstream)	ATTTTCTCCTTAGTTTGTTAGGTAGATAGTAGAAATGGCC	
ΔHdaA-VP1	Pc21g14570 (upstream)	CACACTAAAAGCTCACCGCC	diagnostic PCR for *ΔhdaA*
ΔHdaA-VP2	BleoR	AAGAAGGATTACCTCTAAAC	
ΔHdaA-VP3	Pc21g14570	GGAAAACCTGGCCTTGATGG	
ΔHdaA-VP4	Pc21g14570	TGGCCCGTACAAAGGATTGC	
ΔHdaA-VP5	BleoR	TCGACAGAAGATGATATTGA	
ΔHdaA-VP6	Pc21g14570 (downstream)	TATGGTTGGTGATGTGTCAC	
q12570F	Pc21g12570	AGGGAAATGAATCCAGGTGGC	qPCR of the chrysogine gene cluster
q12570R	Pc21g12570	TAGATGCCGCTTGTTCGGACC	
q12590F	Pc21g12590	TGTGGAGCTCTACGAGGCTG	
q12590R	Pc21g12590	GCTGGCAGGGCTCGTCGGTC	
q12600F	Pc21g12600	CGCCGGTGAGACTTTGATCG	
q12600R	Pc21g12600	TAAGCGTCTAATTTTCATCGC	
q12610F	Pc21g12610	TGCATGCAGCTCCATACGAGC	
q12610R	Pc21g12610	ATAGGTGGAAACAGCTCAGAC	
q12620F	Pc21g12620	TTCGCTGGCTAACTGGTCTCG	
q12620R	Pc21g12620	ATGTGGTAGACGAATTGGAGC	
q12630F	Pc21g12630	GAGCCAACTCTGTTGTCTACG	
q12630R	Pc21g12630	AGGGCAATTTGCCTCATTCTG	
q15420F	Pc21g15420	GTGTCGCTGGCCCTCCATTGG	qPCR of the roquefortine/ meleagrin gene cluster
q15420R	Pc21g15420	GGAGAACACCAGTGAGCACG	
q15430F	Pc21g15430	TGAGATGAGTCCCGGCGAGGC	
q15430R	Pc21g15430	TCCGTTGCGATAACCAAGTCC	
q15440F	Pc21g15440	CAACAGCGGCCTCAACAACGG	
q15440R	Pc21g15440	CTTACTGGCCATGTGAAGCAG	

q15450F	Pc21g15450	GCTCATCAAAGATGCACTACG
q15450R	Pc21g15450	GACACATGGTTATGCAGCGAG
q15460F	Pc21g15460	GCGACATCGTCGGACTGAGAG
q15460R	Pc21g15460	CAATGGAGTCGCGCCACCTGG
q15470F	Pc21g15470	CTTGCGGTGGTTACCAGAGTC
q15470R	Pc21g15470	ACTTTCATCCTCGTACCAACG
q15480F	Pc21g15480	GGATAGTCTCTTGGTGGATGC
q15480R	Pc21g15480	GAGAATGTGAACCGTAGCCG

a. The template genes in strain 1701 were represented with the homologous genes in *P. chrysogenum* Wis 54-1255.

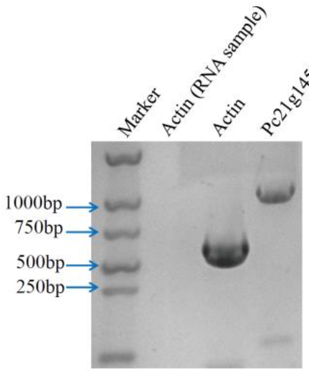

Figure S1 Transcription analysis of potential *hdaA* regulator gene (Pc21g14570) by reverse-transcription PCR. Actin gene of RNA sample and reverse-transcription sample were used as negative and positive controls, respectively.

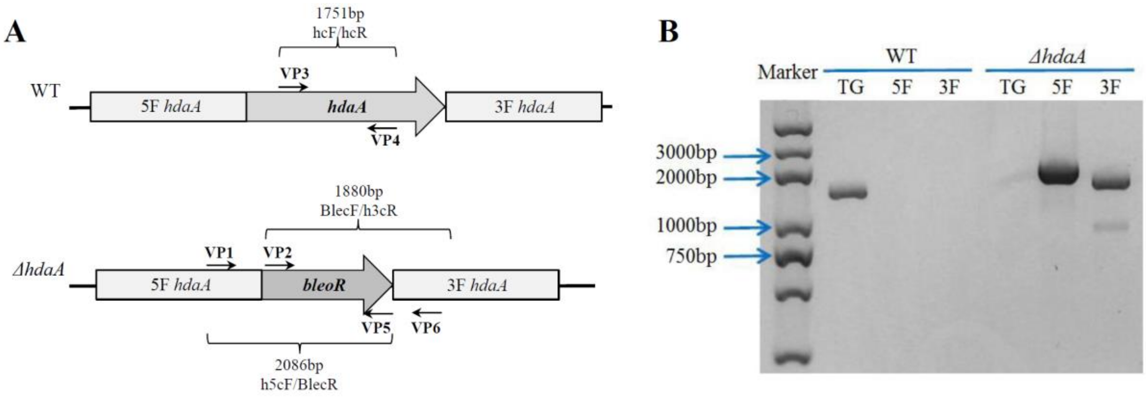

Figure S2 PCR verification of *ΔhdaA* strain. (A) Schematic illustration of diagnostic PCR. Three pairs of primers were used for PCR verification of mutant genotype. The 1.7-kb fragment can only be amplified from the wild-type strain using primers VP3-VP4, but should not appear in *ΔhdaA* mutants. The 2.1-kb and 1.9-kb fragments can be amplified from correct *ΔhdaA* mutants using primers VP1-VP5 and VP2-VP6, respectively, but was absent in the wild-type strain. (B) Genotypic verification of mutant by PCR. Note: Lane TG, using primers VP3-VP4; Lane 5F, using primers VP1-VP5; Lane 3F, using primers VP2-VP6.

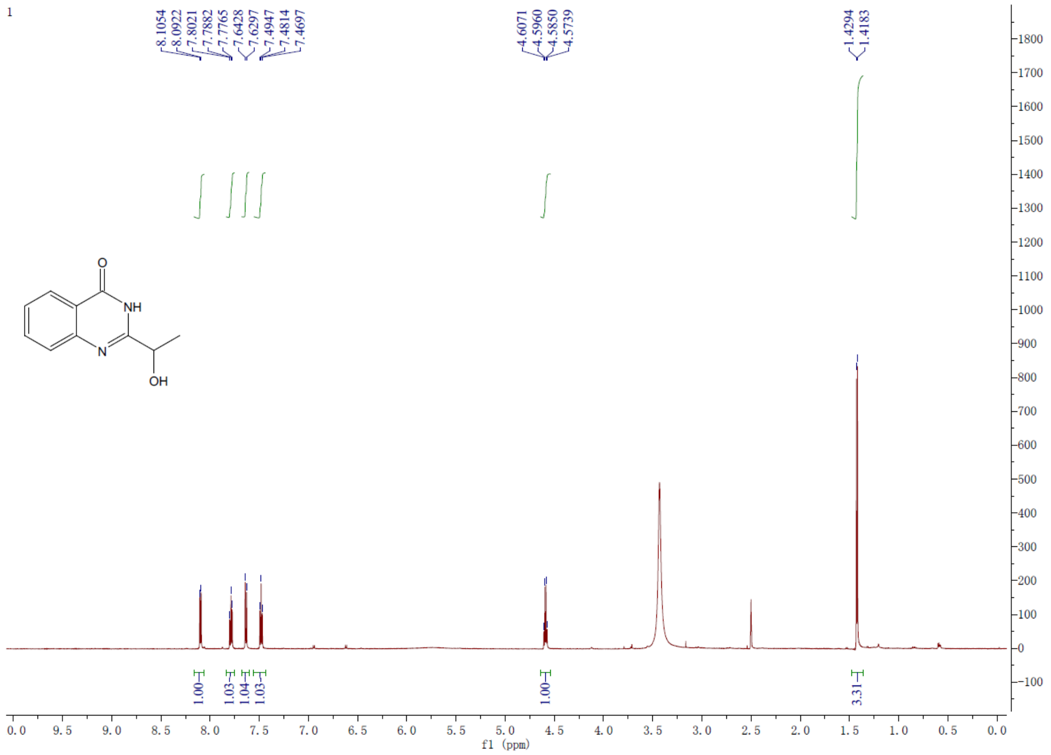

Figure S3 ^{1}H-NMR spectrum of compound **1**.

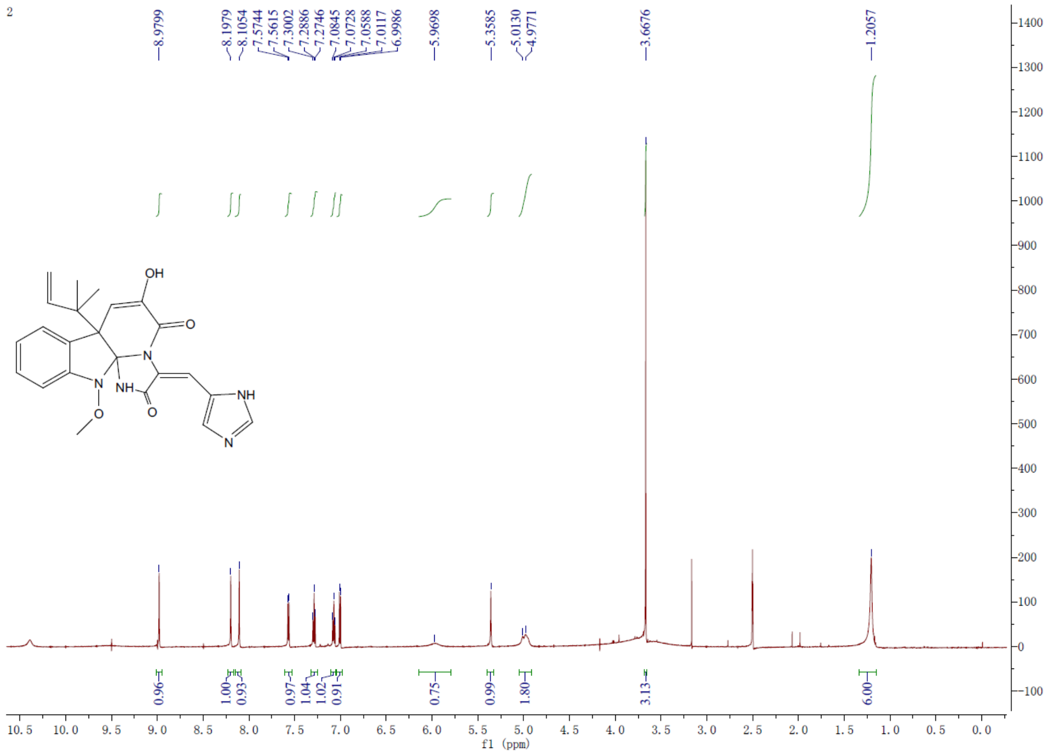

Figure S4 ^{1}H-NMR spectrum of compound **2**.

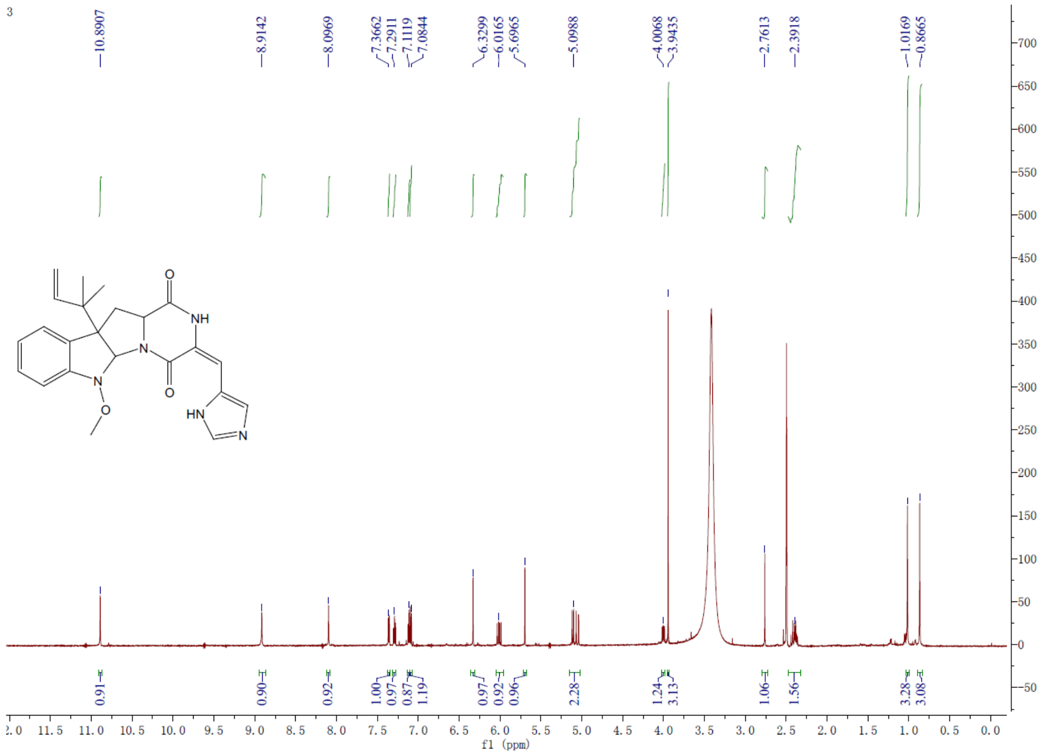

Figure S5 ^{1}H-NMR spectrum of compound **3**.

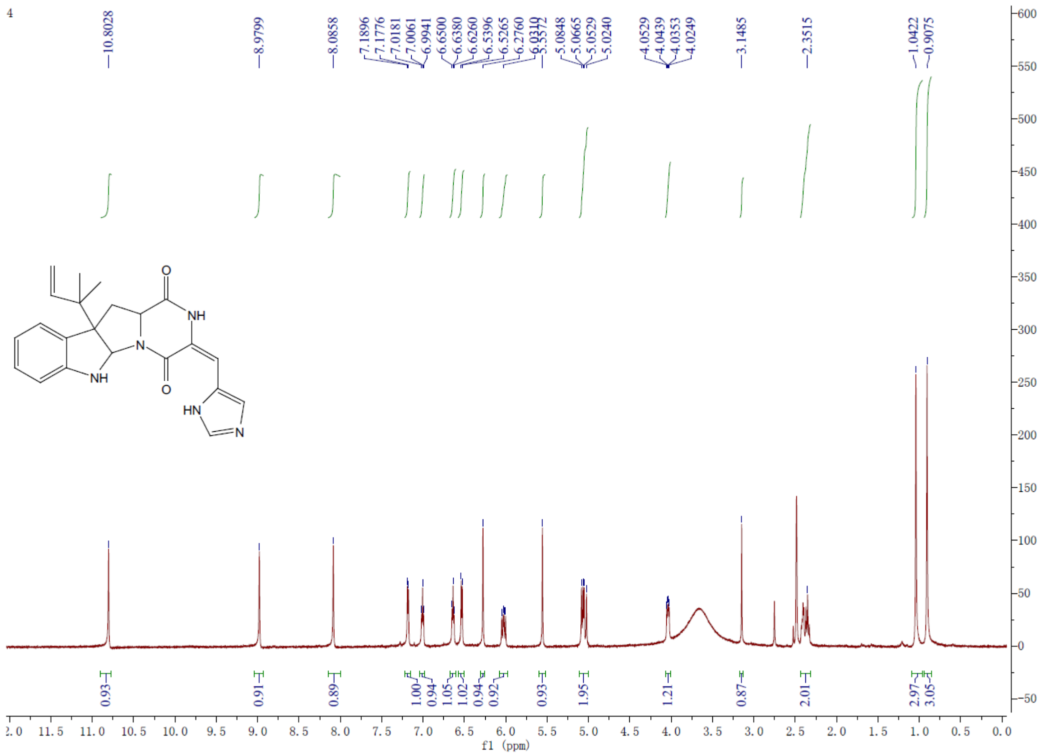

Figure S6 ^{1}H-NMR spectrum of compound **4**.

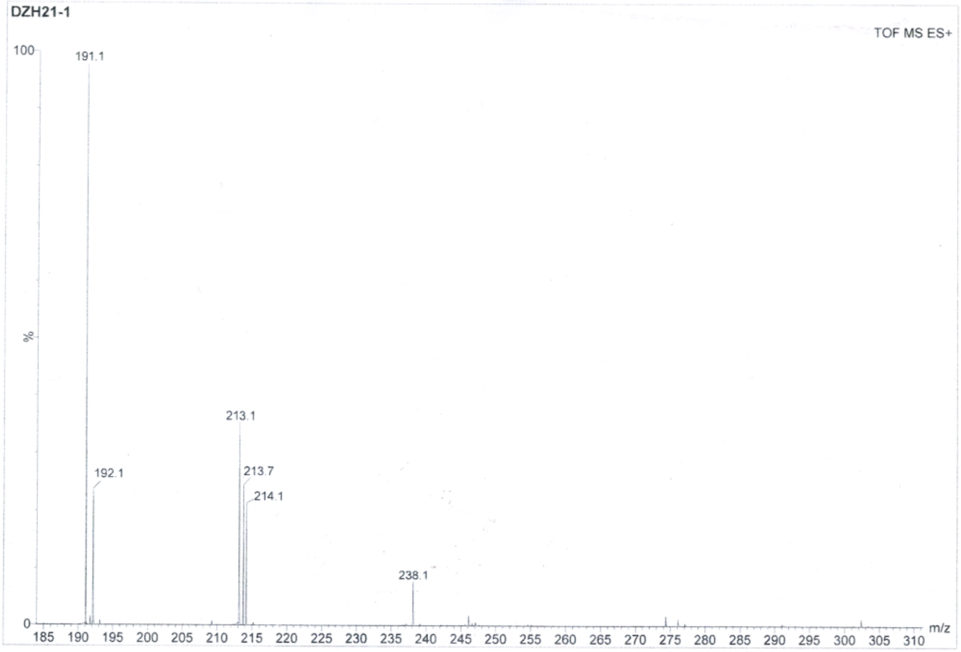

Figure S7 MS spectrum of compound **1**.

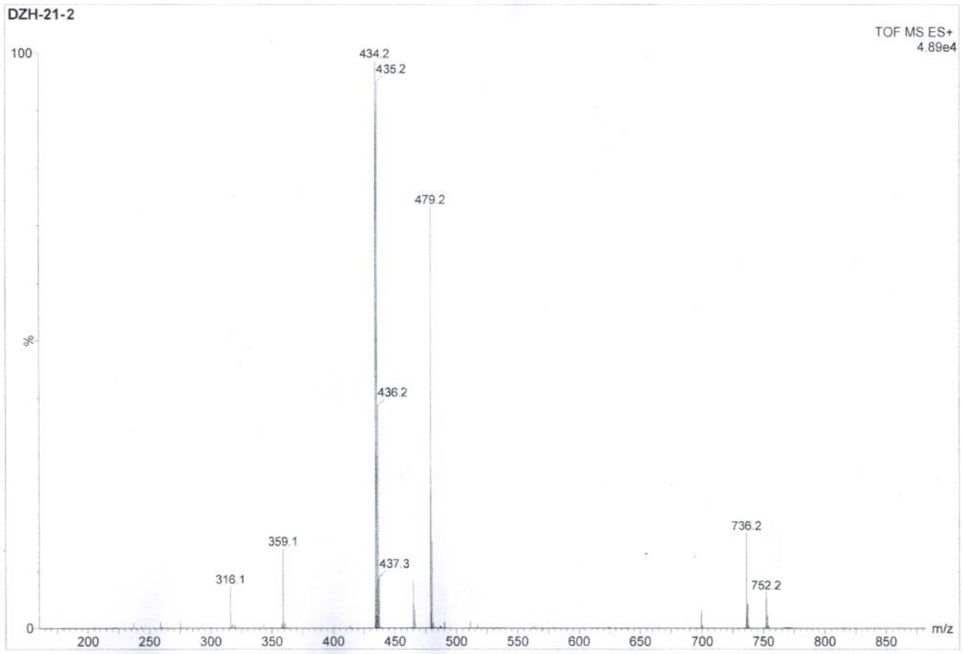

Figure S8 MS spectrum of compound **2**.

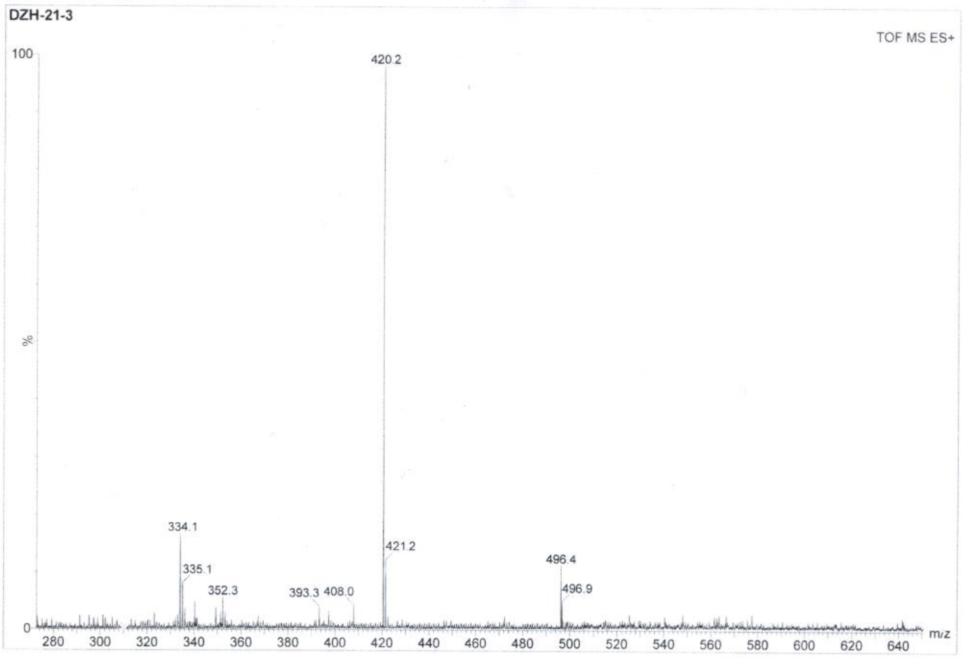

Figure S9 MS spectrum of compound **3**.

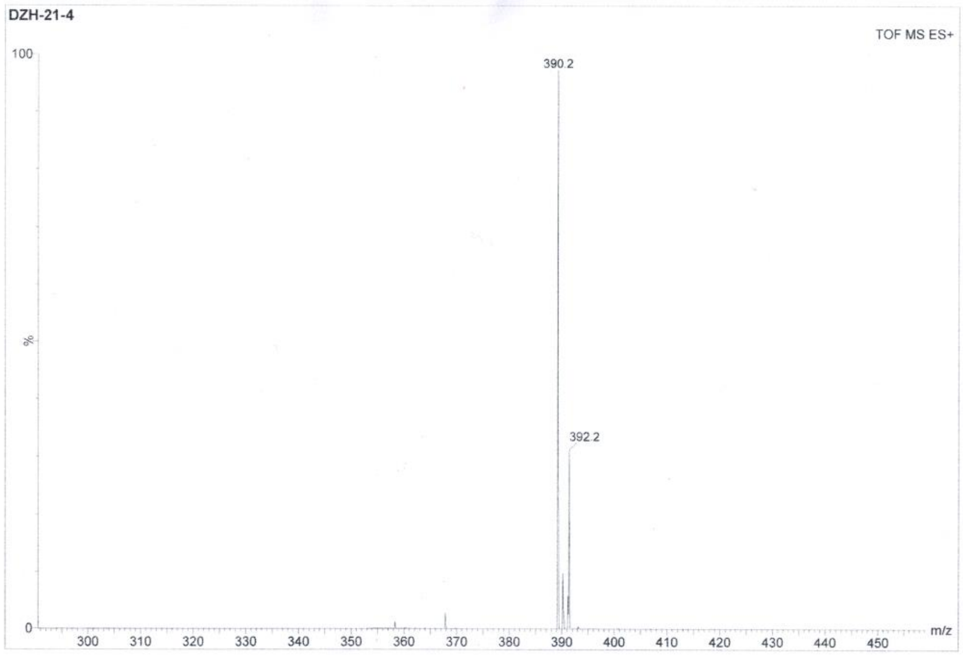

Figure S10 MS spectrum of compound **4**.
